# A CRISPR-Cas9-Based Toolkit for Fast and Precise In Vivo Genetic Engineering of *Bacillus subtilis* Phages

**DOI:** 10.3390/v10050241

**Published:** 2018-05-04

**Authors:** Tobias Schilling, Sascha Dietrich, Michael Hoppert, Robert Hertel

**Affiliations:** 1 Department of Genomic and Applied Microbiology & Göttingen Genomics Laboratory, Institute of Microbiology and Genetics, Georg-August-University Göttingen, 37077 Göttingen, Germany; tschill2@gwdg.de (T.S.); sdietri@gwdg.de (S.D.); 2 Department of General Microbiology, Institute of Microbiology and Genetics, Georg-August-University Göttingen, 37077 Göttingen, Germany; mhopper@gwdg.de

**Keywords:** CutSPR, *Bacillus subtilis* TS01, CRISPR-Cas9, vB_BsuP-Goe1, bacteriophage, phage, virus, artificial

## Abstract

Phages are currently under discussion as a solution for the antibiotic crisis, as they may cure diseases caused by multi-drug-resistant pathogens. However, knowledge of phage biology and genetics is limited, which impedes risk assessment of therapeutic applications. In order to enable advances in phage genetic research, the aim of this work was to create a toolkit for simple and fast genetic engineering of phages recruiting *Bacillus subtilis* as host system. The model organism *B. subtilis* represents a non-pathogenic surrogate of its harmful relatives, such as *Bacillus anthracis* or *Bacillus cereus*. This toolkit comprises the application CutSPR, a bioinformatic tool for rapid primer design, and facilitates the cloning of specific CRISPR-Cas9-based mutagenesis plasmids. The employment of the prophage-free and super-competent *B. subtilis* TS01 strain enables an easy and fast introduction of specific constructs for in vivo phage mutagenesis. Clean gene deletions and a functional clean gene insertion into the genome of the model phage vB_BsuP-Goe1 served as proof of concept and demonstrate reliability and high efficiency. The here presented toolkit allows comprehensive investigation of the diverse phage genetic pool, a better understanding of phage biology, and safe phage applications.

## 1. Introduction

Research on bacterial viruses (phages) is currently one of the most rapidly evolving fields in applied life sciences, mainly due to their potential to act as an alternative to antibiotics for fighting multidrug-resistant bacterial pathogens [1,2]. This was further underlined on the 27th of February 2017, when the World Health Organization (WHO) published a list with 12 top level problematic multidrug-resistant bacteria for the first time [3]. Phages can be a solution and serve as a biological control of multidrug-resistant bacterial pathogens [4]. However, prior to any application it is necessary to ensure the safety of the used phage systems [5]. To assess possible risks for the patient or the environment, profound knowledge of the bacteriophage’s biology and genetics is necessary.

Due to modern genome sequencing technology, a complete phage genome can be obtained shortly after phage isolation. However, analysis of such genomes frequently results in large numbers of putative genes with unknown function or of genes lacking homologues [6]. Classically, a deletion mutant is generated and its phenotype investigated to disclose the function of such a hypothetical gene. While this strategy has been pursued efficiently for prokaryotes and eukaryotes, the ability to modify phage genomes was limited until recently [7]. The introduction of CRISPR-Cas system-based genetic tools was a revolution in many fields of biology [8]. Using this system, it is feasible not only to modify the genome of living cells but also the genomes of their viruses precisely and efficiently [7].

CRISPR-Cas systems naturally serve as a prokaryotic immune system to protect the organism from invading DNA, such as plasmids or viral genomes [9,10,11,12]. They function through a Cas-protein-mediated alignment of a spacer CRISPR RNA (crRNA) to a corresponding target DNA sequence resulting in its cleavage [12]. The association of a specific protospacer adjacent motif (PAM) with the target sequence is essential for the initiation of this process [13]. In the case of a type II CRISPR-Cas system, a trans-acting RNA (tracrRNA) is needed for the processing of pre-cnRNA [14] and the formation of a functional crRNA Cas9 complex [15].

The first reports on phage genetic engineering using CRISPR-Cas technology were presented by Kiro et al. 2014 [16] with *Escherichia coli* as host and Martel and Moineau 2014 [17] with *Streptococcus thermophilus* as host. These are based on host intrinsic CRISPR-Cas systems with individual crRNAs. Recently, two vector-based, *Streptococcus pyogenes*-originated CRISPR-Cas9 systems were published as suitable for phage modification in *E. coli* [18] and *Lactococcus lactis* [19]. Such vector-born systems hold the potential to be used in strains without chromosomal-encoded CRISPR-Cas machinery.

Members of the genus *Bacillus* play an important role for mankind in many ways. On the one hand, strains such as *Bacillus anthracis* and *Bacillus cereus* constitute severe health risks and are a serious threat [20,21]. On the other hand, various *Bacillus* members, such as *Bacillus licheniformis* or *Bacillus subtilis*, are extensively used as biotechnological production strains for enzymes, antibiotics, insecticides, or fine chemicals [22]. *B. subtilis* represents one of the best established model systems, with a wide variety of mutant strains and methods. However, *B. subtilis* does not contain native chromosomal CRISPR-Cas systems. Recently, a vector-based, *S. pyogenes*-originated CRISPR-Cas9 system was published for the genomic modification of *B. subtilis* [23]. In contrast to the vectors used for *E. coli* [18] and *L. lactis* [19], this recruits an sgRNA, which is a functional fusion of a crRNA with a tracrRNA [15,23].

The virulent phage vB_BsuP-Goe1 (Goe1) [24] serves as model phage for this investigation. It reveals *Podoviridae* morphology and is a member of the genus *Phi29virus* [24]. It has a genome of 18,379 bp and is closely related to the well-understood phage phi29 (63.7% global nucleotide similarity). For propagation, it recruits the prophage-free *B. subtilis* ∆6 [25], a derivate of the well-known lab strain *B. subtilis* 168 [26]. Establishing phages of *B. subtilis* as model systems will also lead to a better understanding of phages from *B. anthracis*, *B. cereus*, or *B. licheniformis*, as comparative genomics frequently has revealed similar genomic organization [27].

The aim of this work was to verify the suitability of the *B. subtilis* CRISPR-Cas9 system [23] for genomic modification of phages and its further development. The hereby designed toolkit consists of three main components. The first is the application named CutSPR, which facilitates primer design and assists the preparation of specific mutagenesis vectors. The second is the mutagenesis vector, which is the starting material for the creation of a specific mutagenesis plasmid. The third is the super-competent and prophage-free *B. subtilis* TS01 strain. Its competence can be artificially induced to ensure fast and reliable vector transfer. The prophage-free genetic background excludes unintended interaction of the engineered phage with naturally occurring viral elements in the chromosome. Gene deletions and a functional gene insertion in the genome of phage Goe1 served as proof of concept.

## 2. Materials and Methods

### 2.1. CutSPR

The bioinformatic tool CutSPR was implemented in Python using QT5 for graphical user interface functionality. It internally uses NCBI-BLAST [28], which has to be provided by the user and is not part of the program itself. All parts of the program are licensed under the GPL3 software license and are available as source code on GitHub (https://github.com/sdietri/cutspr) or precompiled executables for Windows, macOS, or Linux operating systems (http://appmibio.uni-goettingen.de/index.php?sec=sw). Detailed information about how CutSPR proceeds on spacer search and primer design is presented in the supplementary material. Melting temperatures for the potential primers are calculated by CutSPR with the “nearest-neighbor” method [29].

### 2.2. Media and Solutions

All media and watery solutions were prepared with water processed with an arium^®^ pro device (Sartorius AG, Göttingen, Germany). If not otherwise stated, *E. coli* and *B. subtilis* strains were cultivated in LB medium (10 g/L tryptone, 10 g/L NaCl, and 5 g/L yeast extract) [30]. LB medium was supplemented with 1.5% (*w*/*v*) Agar-Agar Kobe I (Carl Roth GmbH + Co KG, Karlsruhe, Germany) to prepare solid LB agar plates. Overlay agar for plaque assays was prepared via LB medium supplementation with 0.4% (*w*/*v*) Biozym LE Agarose (Biozym Scientific GmbH, Hessisch Oldendorf, Germany).

### 2.3. Cloning

All *E. coli*-concerning molecular methods were performed according to Sambrook and Russell [31]. Selection for vectors was done with kanamycin (25 µg/mL) in solid and liquid media for *E. coli* and *B. subtilis*. Endonucleases, T4-DNA ligase, FastAP thermosensitive alkaline phosphatase, and Phusion DNA polymerase were applied as recommended by the manufacturer (Thermo Fisher Scientific, Darmstadt, Germany). *E. coli* TOP10 served as cloning strain.

Competent cells of *B. subtilis* ∆6 [25] were prepared as described by Wenzel and Altenbuchner [32] for the introduction of the super-competence cassette. Competent cells of *B. subtilis* TS01 were prepared as described by Rahmer et al. for strain REG19 [33]. Ten milliliters of LB medium in a 100 mL conical flask were inoculated to an OD_600_ of 0.1 with an overnight culture of *B. subtilis* TS01 and incubated at 37 °C for 90 min under vigorous shaking. Subsequently, the culture was supplemented with d-mannitol to a final concentration of 0.5% (*w*/*v*) and cultured for a further 90 min. Cells were pelleted at 3500× *g* and 4 °C for 5 min, then washed twice with 10 mL of ice cold LB medium. The final cell suspension was set to an OD_600_ of 0.5. Aliquots of 1 mL were used for transformation. Competent *B. subtilis* TS01 cells were cryo-conserved at −80 °C after adjustment with glycerol to a final concentration of 20% (*v*/*v*). For transformation, cells were thawed on ice. Cells were transformed by mixing with 100 ng of DNA in a volume of 20 µL in a 15 mL sterile tube, followed by incubation for 60 min at 30 °C on a roller drum (RM5 Type 348, Hecht-Assistant, Sondheim v. d. Rhön, Germany) and subsequent plating on selective LB plates. The primers used in this study are listed in Table S1 presented in the supplementary material.

### 2.4. Primer Design for the Super-Competence Cassette

Primers for the super-competence cassette were designed manually but following the logic presented for CutSPR.

### 2.5. Cloning of the sgRNA Targeting Genes Goe1_c00180 and Goe1_c00030

For sgRNA construction targeting Goe1_c00180, 10 µL of primers TS015 and TS016 (5 µM) were mixed and incubated for 30 min at room temperature. One microliter of this hybridization was ligated with 40 ng of BsaI, digested, and via gel electrophorese purified pJOE8999 vector (NucleoSpin^®^ Gel and PCR Clean-up (Düren, Germany)) using T4-DNA-Ligase. It was then transformed into chemically competent *E. coli* cells and plated onto selective LB agar plates. Clones were initially verified via restriction analysis with the endonuclease EcoRI. In the case of a correct restriction pattern (~3 kb and 4 kb and no 200 bp), clones were further verified via Sanger sequencing (SeqLab-Microsynth, Göttingen, Germany) using primer RH003.

For sgRNA construction targeting Goe1_c00030, pJOE8999 was digested with BsaI, dephosphorylated, and purified with the NucleoSpin^®^ Gel and PCR Clean-up (Düren, Germany). An amount of 0.016 pmol of the prepared pJOE8999 were mixed with 0.08 pmol primer TS047, mixed with the NEBuilder^®^ HiFi DNA Assembly Master Mix (New England Biolabs GmbH, Frankfurt, Germany), and incubated for ligation as recommended by the manufacturer. Transformation and construct verification was performed as described above. It resulted in the pTS009 vector.

### 2.6. Recombination Cassette Cloning

Flanks were amplified via PCR with specific primers for the genetic material, verified via TAE agarose gel electrophoresis, and purified with NucleoSpin^®^ Gel and PCR Clean-up (Düren, Germany). DNA concentration was determined and adjusted to 0.1 ng/µL for each DNA fragment. Ten microliters of each fragment were mixed and 1 µL of this mixture used as template for a fusion PCR with the external primers. The resulting PCR product was verified, purified as described above, and processed with the SfiI endonuclease. It was cloned into a SfiI-digested and dephosporylated pJOE8999 vector. Positive clones were verified via control digestion with SfiI endonuclease followed by TAE agarose gel electrophoreses. Sequences were verified via Sanger sequencing (SeqLab-Microsynth, Göttingen, Germany) using primer RH001, primer RH002, and if necessary further primer used for construction.

Cloning of the recombination cassette for Goe1_c00030 deletion was performed as described for pTS003 using SfiI restriction sites and vector pTS009. It resulted in the vector pTS010.

### 2.7. Construction of Mutagenesis Vectors

The construction of specific mutagenesis vectors from an empty vector was performed as described for pJOE8999 by Altenbuchner [23] and also served as base for the working instructions given by the CutSPR application.

The recombination cassettes consisted of Flank A with 712 bp and Flank B with 709 bp. In the case of the deletion cassette with a size of 1421 bp, 762 bp of the head fiber gene (Goe1_c00180) were deleted by joining the starting (ATG) triplet with the last three triplets of the gene including the stop codon. In the case of the insertion cassette with a final size of 3428 bp, the *bgaB* gene with 2013 bp was placed between the start and the stop codon of the head fiber gene. Sequences of the final vectors were verified via Sanger sequencing (SeqLab-Microsynth, Göttingen, Germany) using primers RH001 and RH002 for the recombination cassette. In the case of the insertion cassette, the *bgaB* gene was additionally sequenced with TS030 and TS031. Primer RH003 served for sgRNA sequencing.

### 2.8. Plaque Assay and Analysis

Infection was performed by mixing 100 µL of the host overnight culture with 100 µL of a phage suspension. After incubating for 5 min, 2 mL of pre-warmed (50 °C) overlay agar were added, mixed, and placed on solid LB agar plates. Overlay plates were incubated for 16 h at 30 °C. Plaques of interest were picked with a sterile toothpick and placed into 500 µL sterile LB medium for 10 min. The so-prepared phage suspension was stored at 4 °C. For further separation, dilutions were made with sterile LB medium and used for plaque assay as described above. Verification of specific plaques was done by PCR amplification with a specific primer pair and 1 µL of the initial phage suspension as template. PCR products were analysed by electrophoresis in 0.8% TAE agarose gels in combination with ethidium bromide staining [31]. PCR products of interest were purified with the NucleoSpin^®^ Gel and PCR Clean-up kit following the instructions of the manufacturer (MACHEREY-NAGEL, Düren, Germany). Sanger sequencing was performed by SeqLab-Microsynth (Göttingen, Germany).

### 2.9. Specific Plaque Assay

For genetic modification of phage Goe1, an adapted plaque assay was employed. A host strain harboring a specific mutagenesis vector was used for infection. Cells embedded in overlay agar were plated on LB medium plates supplemented with d-mannose (0.2% *w*/*v*). The replication ability rate of phages after Cas9 interference ranged from ~0.25% to ~0.35% on both targets. Consequently, an infection load of about 10,000 plaque forming units was used to receive 25 to 35 phage plaques for the identification of the desired mutant. Resulting plaques were picked and used in a standard plaque assay with a vector-free host to achieve further purification of the isolates. A standard plaque assay was performed on LB agar plates supplemented with X-Gal (50 µg/mL) to verify functional expression of the *bgaB* gene during phage propagation.

### 2.10. Phage Propagation in Liquid Culture

To generate a concentrated phage suspension, phage amplification in liquid culture was performed. Four milliliters of LB medium were inoculated with an overnight culture of the host strain to an OD_600_ of 0.05 and incubated for 3 h at 30 °C under vigorous shaking. The culture was then infected with 500 µL of phage suspension initially prepared from a freshly picked plaque. The infected culture was further incubated until it was totally clear. This total lysate was passed through a 0.45 µm pore size filter (Filtropur S 0.45, SARSTEDT, Nümbrecht, Germany) and stored at 4 °C.

### 2.11. Transmission Electron Microscopy

Negative staining, transmission electron microscopy, and micrograph processing were performed as previously described [34]. Uranyl acetate dissolved in pure water (4%, *w*/*v*) served as staining solution. Negative staining was performed with carbon films deposited on mica essentially as described by Valentine et al. 1968 [35] with modifications as described by Hoppert and Holzenburg 1998 [36]. Electron microscopy was carried out with a Jeol 1011 electron microscope (Eching, Munich, Germany) in combination with a Gatan Orius 4 K camera and the Gatan 314 Digital Micrograph software package (Gatan GmbH, Munich, Germany).

### 2.12. Pairwise Sequence Alignment

Pairwise Sequence Alignments of nucleotide or protein sequences were performed with the web-based EMBL-EBI services with default parameters and the Needleman–Wunsch algorithm (https://www.ebi.ac.uk/Tools/emboss/).

## 3. Results

### 3.1. CutSPR

For precise and efficient phage genetic engineering, some initial procedures are necessary. On the one hand, a 20 bp unique spacer sequence is needed to create a specific sgRNA, which is essential for the Cas9 nuclease to cleave the addressed target. On the other hand, a recombination cassette is required to ensure repair of the Cas9-mediated double strand break via homologous recombination, leading to the desired genotype. Primers for the preparation of both elements can be designed manually, which is time-consuming as many aspects need to be considered, such as primer extensions for cloning or a PCR base fusion of DNA fragments for the construction of the recombination cassette.

In order to support the primer design process, the bioinformatic tool CutSPR was designed. It generates custom primers based on experiment-specific requirements. For the sgRNA design, the sequence of the deletion target can be pasted into the user interface. Genetic background information of the bacterial host and the targeted virus can be added to the program as GenBank, EMBL, and FASTA files to ensure the specificity of the sgRNA to its target. The number of background sequence files is not limited, which enables additional consideration of natural or artificial plasmids. By initiating the search for a specific spacer sequence, CutSPR first identifies all potential protospacer adjacent motifs (PAMs) on the positive and negative strand of the target sequence. Based on this, the specific 20 bp regions are defined. To identify the best candidate, all regions of interest are matched against all background sequence data via nucleotide BLAST [28]. The first 100% hit, representing the self-hit, is ignored and all other hits are ranked and listed beginning from the most individual. A user-selected sequence is then used for primer design, including “sticky”-5′-extensions to enable cloning into a BsaI-digested pJOE8999 vector, thereby creating a specific sgRNA. Primers for the creation of a specific recombination cassette are designed automatically. It is possible to define the targeted genome as circular or define the desired size of the homologous regions. The desired size of the homologous regions is of utmost importance in the case of problematic elements, such as small toxic protein-coding regions, which should be excluded from the recombination cassette. In case an insertion is desired as a replacement for the deleted region, it is possible to enter the insert sequence into the designated field. The insert will then be considered and an additional set of primers will be generated to implement the insert into the recombination cassette. Finally, the tool lists all obtained primers and provides detailed working instructions, which lead to the creation of a specific CRISPR-Cas9-based system.

### 3.2. B. Subtilis TS01

**Figure 1:**
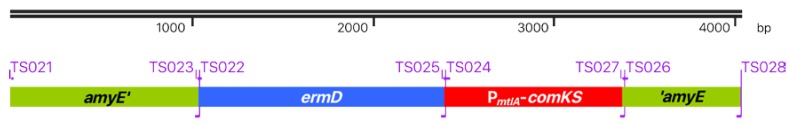
*ermD*-P*_mtlA_*-*comKS* super-competence cassette. The modified super-competence cassette consists of the *amyE* homolog regions *amyE*’ and ’*amyE* for recombination with the genome of *Bacillus subtilis* Δ6, the *ermD* erythromycin selection marker, and the P*_mtlA_-comKS* competence cassette with the genes *comK* and *comS* under the control of the d-mannitol-inducible P*_mtlA_* promotor. Primers used for the construction are shown on the border of each element.

CRISPR-Cas-based genetic work with phages implies work with a bacterial host, which provides the metabolic resources for phage replication and engineering. Such a host should match two criteria to enable high-throughput engineering. Firstly, vector introduction should be convenient and fast, and secondly, the host strain should be prophage-free to avoid interaction of the engineered phage with prophages from the host genome. To create such a strain, we decided to combine the properties of already existing *Bacillus* strains. The d-mannitol inducible super-competence cassette of *B. subtilis* REG19 [33] was transferred to the prophage-free genomic background of *B. subtilis* Δ6 [25]. To achieve this goal, the P*_mtlA_*-*comKS* competence cassette from *B. subtilis* REG19 was combined with the erythromycin resistance marker *ermD* from *B. licheniforims* 9945A [37]. This modified super-competence cassette was fused via PCR to homologous regions of the *amyE* gene (see Figure 1) and consequently integrated into the *amyE* locus of *B. subtilis* Δ6.

The genotype of the new strain was verified via Sanger sequencing of the respective region. The phenotype was verified via transformation of the pJOE8999 plasmid. An average transformation efficiency rate of 1 clone per 100 ng DNA was observed for *B. subtilis* Δ6, 62 clones for *B. subtilis* REG19, and 426 clones for *B. subtilis* TS01 (see Table 1). These results verify the functionality of the introduced inducible competence-cassette and the suitability of the strain for transformation with a plasmid-based vector system. Competent cells of *B. subtilis* TS01 were successfully cryo-conserved at −80 °C and used for subsequent transformations. Cryo-conservation of the competent cells consistently resulted in a drop of transformation efficiency by about one order of magnitude. However, competence remained sufficient for successful plasmid introduction.

### 3.3. Genome Modification of vB_BsuP-Goe1

#### 3.3.1. Gene Deletion

To verify whether the pJOE8999 vector system [23] is suitable for in vivo genetic engineering of virulent phages, a proof of concept experiment with the smallest known *B. subtilis* phage Goe1 [24] was performed. The gene Goe1_c00180 coding for the head fiber protein was selected as target. Its homolog of phage phi29 [38] was described as non-essential [39,40]. The required sgRNA was constructed via ligation of a primer-pair hybridization (TS015 and TS016) into the pJOE8999 vector, which had previously been digested with the BsaI endonuclease. In this manner, vector pTS002 was constructed. Using Goe1 chromosomal DNA as PCR template, a recombination cassette was constructed (primers TS010, TS011, TS013, and TS014) and cloned into pTS002 via the SfiI restriction sites. This resulted in the pTS003 vector, which was transformed into *B. subtilis* TS01. The generated strain *B. subtilis* TS01pTS003 was infected with the wild-type phage Goe1. From the resulting plaques, nine were selected for a further plaque assay to purify potential mutants. Finally, 36 phage plaques were picked and their genotype investigated via PCR (Primers TS013 and TS014). The size-separated PCR products revealed fragments of different length. Two clones revealed double bands indicating a mixed phage suspension, and 19 clones showed slightly smaller bands compared to the wild-type situation (~2.2 kbp), which may be a product of a non-homologous end-joining (NHEJ) after a double strand break mediated by the Cas9 nuclease. Fifteen clones showed bands indicative of the desired deletion mutants in which the Cas9-mediated double strand break was successfully repaired through recombination with the vector-provided recombination cassette.

Sanger sequencing of the PCR products from potentially correct deletion mutants verified precise gene removal. Thus, the CRISPR-Cas9-based vector pJOE8999 is highly effective when used for gene deletion in the genome of a virulent phage. With this procedure, an efficiency rate of up to 40% could be achieved with regard to the investigated plaques. Moreover, the genome of Goe1 could be reduced by around 4% (now 17,617 bp) making it the smallest artificial *B. subtilis* phage.

**Figure 2:**
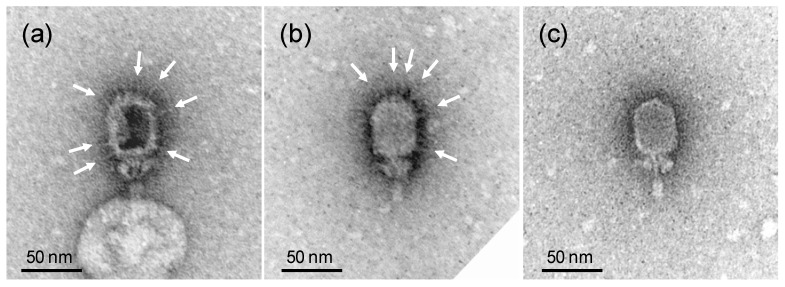
Morphology of Goe1 and Goe1 Δc00180. Transmission electron micrographs of negatively stained samples. (**a**,**b**) illustrate the wild-type strain Goe1 with head fibers indicated with white arrows; (**c**) shows a virion of the Goe1 Δc00180 mutant strain without head fibers.

As the removed gene Goe1_c00180 codes for a head fiber protein, a head fiber deficient morphological phenotype was expected. To verify this, the morphology of the modified phage was investigated via transmission electron microscopy revealing virions free of head fibers (see Figure 2).

To further verify the reliability of this system, the gene Goe1_c00030 coding for a hypothetical protein was chosen as an additional deletion target. The deletion vector pTS010 was constructed and applied like pTS003. Ten clones were picked from the initial mutagenesis experiment and faced further separation on a plasmid-free *B. subtilis* TS01. Four of 40 clones revealed the desired genotype during PCR-based verification, which was confirmed on two selected mutants via Sanger sequencing. The so-constructed derivate of phage Goe1, lacking the hypothetical gene Goe1_c00030, revealed a genome shortened by 168 bp, resulting in a total length of 18,211 bp. Moreover, this experiment proved that the respective gene is not essential.

#### 3.3.2. Gene Insertion

After successful gene deletion, the feasibility of gene insertion was tested with pJOE8999. For this, the *bgaB* gene, coding for a thermostable beta-galactosidase from *Bacillus stearothermophilus* [41], was amplified from the vector pKVM1 [42] (Primer TS030, TS031) and fused to the surrounding region of the head fiber gene Goe1_c00180 (Primer TS013, TS014, TS029, TS030, TS031, TS032). The coding region of *bgaB* (2013 bp) was placed in frame between the start and stop codon of Goe1_c00180. This recombination cassette (3428 bp) was processed with the SfiI endonuclease and ligated into the vector pTS002. The resulting plasmid pTS004 was transformed into *B. subtilis* TS01, thereby generating the strain *B. subtilis* TS01pTS004. This strain was infected with the wild-type Goe1 and potential insertion mutants were selected as described previously for the deletion mutants. In total, 40 potential Goe1 Δc00180::*bgaB* clones were picked and analyzed analogously to the deletion mutants. Seventeen clones revealed a wild-type-like band, 21 clones showed bands slightly smaller than the wild-type, suggesting a non-homologous end-joining event, and 2 clones showed bands corresponding to correct insertion of the *bgaB* gene. These phage mutants revealed a 7% larger genome with a total size of 19,636 bp and thereby exceeded the genome size of phi29 (19,282 bp) [38] by 354 bp.

**Figure 3:**
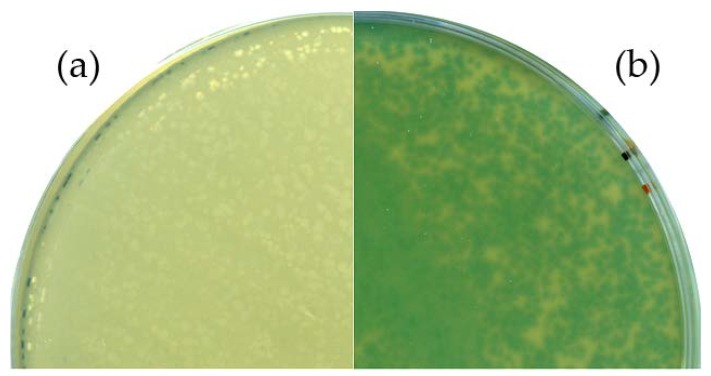
Phenotypic analysis of the Goe1 Δc00180::*bgaB* mutant. For both experiments, X-Gal-supplemented agar plates were used. *B. subtilis* TS01 was infected with (**a**) the Goe1 wild-type; and (**b**) Goe1 Δc00180::*bgaB*. The blue color in the case of the *bgaB* insertion mutant indicates β-galactosidase activity, while no activity could be observed for the control experiment with the Goe1 wild-type.

Sanger sequencing of the respective region confirmed the desired genotype. An X-Gal plaque assay was performed to verify the functional expression of the thermostable beta-galactosidase during virus propagation. An infection experiment with the wild-type phage served as control. Plates of the control experiment showed no color change, whereas a distinct blue coloring was observed in the Goe1 Δc00180::*bgaB* plaques (see Figure 3), indicating the functionality of the enzyme. Thus, the functional expression of the inserted gene and the phenotype of the new mutant phage were confirmed.

In summary, even though the efficiency of the *bgaB* insertion was strongly reduced (5%) compared to the gene deletion experiment (see Table 2), the results proved that gene introductions are feasible using pJOE8999.

## 4. Discussion

Construction of specific virulent phage mutants of *B. subtilis* has been a daunting task so far. Hundreds of random mutants were necessary to enable investigation of the head fiber gene from phi29 and its importance for the virus [39]. With the here presented CRISPR-Cas9-based toolkit, the orthologous gene from Goe1 (~64% global protein similarity) was rapidly deleted and even replaced by a bacterial beta-galactosidase gene. The possibility to introduce foreign DNA in this manner is of special interest. It enables us to target the insertion on a subsequent mutagenesis and reintroduces the deleted gene with modifications, which would be almost impossible with a direct approach. Such modifications could be point mutations for codon adaptation or stop codon introduction for example. The appearance of potential NHEJ events could be explained by the genomic presence of *ku* and *ligD*, the intrinsic NHEJ system of *B. subtilis* [43]. Similar effects were reported in other prokaryotes after Cas9 cleavage [44,45,46]. Deletion of *ku* and *ligD* from the genome of *B. subtilis* TS01 could further optimize the process of phage genetic engineering and may increase the desired mutant rate per screened plaque.

The bioinformatic tool CutSPR enables fast and precise primer design for pJOE8999 modification into a specific mutagenesis vector. In contrast to many similar solutions listed on the addgene website (https://www.addgene.org/crispr/reference/), CutSPR can run on a local computer, which ensures privacy and the usage of unpublished and sensitive genetic material. Spacer identification is focused on a given target and it considers the genetic background of the bacterial host and the virus to reduce potential off-target effects. CutSPR creates primers for sgRNA cloning and for the design of the recombination cassette. Furthermore, it provides detailed cloning instructions. Even though CutSPR is optimized for the pJOE8999 vector, it may also be useful for other *S. pyogenes* CRISPR-Cas9-based applications and beyond. Critical parts, such as spacer length, primer extension, or the PAM, can be modified manually making the output compatible with potential further cloning strategies or other types of CRISPR-Cas systems. Moreover, its assistance with the generation of deletion or insertion recombination cassettes can be helpful for other genetic modification systems based on homologous recombination [33,42].

*B. subtilis* TS01 is predestinated to serve as a model organism for the genetic engineering of phages. The chromosomal absence of phage-related elements prevents any undesired recombination with the engineered virus. Its artificial genetic competence enables simple and rapid DNA transfer. This process takes about 90 min, including 60 min of incubation with cryo-conserved cells. The super-competence phenotype of *B. subtilis* TS01 was more pronounced compared to *B. subtilis* REG19, which may originate from its prophage-free genetic background. Despite all of the advantages of *B. subtilis* TS01, the vector-based nature of the mutagenesis system is not limited to this strain. It also permits usage in further strains of the *B. subtilis* species, thereby enabling investigation of phages which do not infect derivatives of *B. subtilis* 168, such as *B. subtilis* ∆6 or TS01.

Experiments analogous to the deletion of the head fiber in Goe1 will allow the study of many viral genes via phenotype investigation of deletion mutants. The ability to insert genes will enable complementation tests examining if gene variants serve a similar purpose. The combination of recombinant phages and the broad variety of methods available for *Bacillus* will increase the understanding of in-host processes before lysis. Thus, co-purification experiments of tagged and cross-linked proteins [47] hold the potential to increase our understanding of viral protein interaction networks during viral reproduction.

Taken together, this work demonstrates that phages of *Bacillus subtilis* can be modified with a CRISPR-Cas9-based mutagenesis system. Together with the application CutSPR and the *B. subtilis* TS01 host, this system may initiate a revival of *B. subtilis* as model organism for phage genetics.

## Figures and Tables

**Table 1:** Transformation efficiency of the *B. subtilis* strains Δ6, REG19, and TS01.

Experiment	∆6	REG19	TS01
1	0	11	120
2	0	64	714
3	4	111	445
4	no data	no data	599
average	133	6200	42,633

All three strains were treated equally as described in Materials and Methods for *B. subtilis* TS01. Transformation efficiency was determined with 1 mL (OD_600_ 0.5) of freshly prepared cells with 100 ng of pJOE8999 vector. Cells spread on selective plates were incubated at 30 °C for 16 h.

**Table 2:** Modification efficiency of vB_BsuP-Goe1.

Modification	Verified Plaques	Correct Mutants	Efficiency
Goe1 Δc00180	36	15	40%
Goe1 Δc00030	40	4	10%
Goe1 Δc00180::bgaB	40	2	5%

## Supplementary Materials

Supplementary materials can be found at http://www.mdpi.com/1999-4915/10/5/241/s1.
