# Supplementary material for: A CRISPR-Cas9-Based Toolkit for Fast and Precise In Vivo Genetic Engineering of Bacillus subtilis Phages
Source: Viruses. 2018 May 4;10(5):241. doi: 10.3390/v10050241 (PMC5977234; doi:10.3390/v10050241)
Supplement: Supplementary file 1 [file viruses-10-00241-s001.pdf]

viruses

MDPI

Supplementary Material

A CRISPR-Cas9-Based Toolkit for Fast and Precise In Vivo Genetic Engineering of *Bacillus subtilis* Phages

Tobias Schilling[1], Sascha Dietrich[1], Michael Hoppert[2], and Robert Hertel[1,*]

1 Department of Genomic and Applied Microbiology & Göttingen Genomics Laboratory, Institute of Microbiology and Genetics, Georg-August-University Göttingen,37077 Göttingen, Germany
2 Department of General Microbiology, Institute of Microbiology and Genetics, Georg-August-University Göttingen,37077 Göttingen, Germany
* Correspondence: rhertel@gwdg.de; Tel.: +49-551-39-91120

2.1.1 Spacer search by CutSPR

CutSPR has to be supplied with background genetic information, such as the genome of the viral bacterial host, plasmids, cloning host, and the viral genome of interest, in FASTA, EMBL, or GenBank format. The sequence targeted for deletion has to be passed into the provided interface. By default, the protospacer adjacent motif (PAM) is set to NGG and the spacer length to 20 bp. By pressing the button "Search Spacer Sequence", CutSPR identifies all existing PAMs in the targeted sequence and extracts the associated protospacers. All candidates are compared with the background genetic material via nucleotide BLAST [28] to search for secondary hits. Hits lacking a PAM at the appropriate position are discarded. Remaining sequence hits are verified for the presence of a potential seed sequence (10xN-**8 bp-seed**-NN-PAM) [13]. This may be of importance as the initial annealing of a seed sequence with a protospacer leads to the stabilization of the Cas9, sgRNA, and protospacer complex. Although such an annealing with a seed sequence is only insufficient for target cleavage, its frequent appearance may keep the Cas9 sgRNA complex away from its designated locus. Finally, CutSPR lists all suitable spacers and presents their sequence, GC-content, percent similarity to a second hit on the genetic background, and the count of potential seeds. It orders the hits based on their uniqueness (up to 50% green, 50–70% yellow, >70% orange) followed by the number of potential seeds. To proceed with primer design, a spacer sequence needs to be selected by the user.

2.1.2 Primer design by CutSPR

The introduced deletion target is identified in the background sequence material via nucleotide BLAST and the surrounding sequence used for the creation of the deletion or insertion (≥50 bp) cassette. Melting temperatures for the potential primers are calculated with the "nearest-neighbor" method [29]. At the fusion position, a sequence of 23 bp is selected for each flank and verified for its melting temperature. In the case where it is <55 °C, the sequence is extended until it is ≥55°C. To ensure a mutual annealing of flanks during fusion via PCR, primers are extended with 12 bases of the corresponding fragment and the melting temperature is calculated. Overhangs are extended or shortened until they reach a melting temperature range between 27 and 31°C.

The search for external primers of the flanks is initiated 30 bases outside the user-defined flank size (700 bp default). Initially, 23 bases are picked and their melting temperature determined. If the melting temperature is <56°C, the primer is extended until the melting temperature reaches ≥56°C. This sequence is then compared with the genetic background via nucleotide BLAST. In the case where a second 100% hit is determined, the primer sequence is discarded. If not, the melting temperature on the second-best hit is verified. In the case where it is ≥40 °C, the primer is discarded and a new round is initiated one base closer to the flank border until a sequence fits the mentioned requirements or the minimum flank size is violated, in which case CutSPR requests new input.

Table S1. Primers used in this study.

Name	Sequence 5′→ 3′	Purpose
TS010	cacctctatagaatCATTACGCTTGCGCACCTCC	Flank A for Goe1_c00180 deletions cassette
TS011	gcgtaatgATTCTATAGAGGTGAAATTTCATGGC	Flank B for Goe1_c00180 deletion cassette
TS013	taggatccggccaacgaggccTTAAGAAAGCACGAGCAACAGCG	Flank A for Goe1_c00180 deletions and insertion cassette
TS014	taggatccggccttattggccCAACTTGCACTTCGTTGCTACC	Flank B for Goe1_c00180 deletion and insertion cassette
TS015	tacgTTCAACAAGTGAAGACAATA	sgRNA cloning to target Goe1_c00180
TS016	aaacTATTGTCTTCACTTGTTGAA	sgRNA cloning to target Goe1_c00180
TS021	TCAATGGGGAAGAGAACCGCTTAAG	amyE′ from *B. s.* Δ6 for s.-competence-cassette
TS022	ccctgtcaacgtcGACATGGATGAGCGATGATG	amyE′ from *B. s.* Δ6 for s.-competence-cassette
TS023	ccatgtcgacGTTGACAGGGACATCTGAATCC	*ermD* from *B. l.* 9945A for s.-competence-cassette
TS024	aagctagcggCTGCGATATTCGTAAGGAGAAGAAAATTC	*ermD* from *B. l.* 9945A for s.-competence-cassette
TS025	aatatcgcagCCGCTAGCTTTTTATTTTTAAAAAATTGTCAC	P_{mtlA}-*comKS* from *B. s* REG19 for s.-competence-cassette
TS026	tagtacataaGGAGGATTTCGTGCCGGTTG	P_{mtlA}-*comKS* from *B. s* REG19 for s.-competence-cassette
TS027	gaaatcctccTTATGTACTATTTCGATCAGACCAGTTTTTAATTTG	′amyE from *B. s.* Δ6 for s.-competence-cassette
TS028	AACAAAATTCTCCAGTCTTCACATCG	′amyE from *B. s.* Δ6 for s.-competence-cassette
TS029	CATTACGCTTGCGCACCTCC	Flank A insertion cassette
TS030	ggaggtgcgcaagcgtaatgaATGTGTTATCCTCAATTTGTTACGG	*bgaB* insertion cassette
TS031	catgaaatttcacctctaatggtggtggtgatgatgAACCTTCCCGGCTTCATCATG	*bgaB* insertion cassette
TS032	ACCACCATTAGAGGTGAAATTTCATGGCATATGTG	Flank B insertion cassette
RH001	AGCTTAGGCCCAGTCGAAAG	pJOE8999 sequencing primer for recombination cassette
RH002	CAGCTAGGAGGTGACTGAAG	pJOE8999 sequencing primer for recombination cassette
RH003	ACCGAGCGTTCTGAACAAATCC	pJOE8999 sequencing primer for sgRNA
TS047	agagaattgagtaaaatgtacctacgCTATCAAATCTGTAACCTCTGTTTTagagctagaa atagcaagttaaaat	sgRNA cloning to target Goe1_c00030
TS048	cgactcactatagggtcgacggccaacgaggccAAAGTAAGCCCCCACCCACTTTC	Flank A for Goe1_c00030 deletion cassette
TS049	aattgaatctggtgaaagaaatatgagATGAATGAATTTATGGTATAATTGTCGTAG ACAAGG	Flank A for Goe1_c00030 deletion cassette
TS050	gtctacgacaattataccataaattcattcATCTCATATTTCTTTCACCAGATTCAATTTA ACGA	Flank B for Goe1_c00030 deletion cassette
TS051	gacttcatataaaatctacttctaataaagaattagatctttggccttattggccGAGTGTTCTCCTGAT GAAGCTACGA	Flank B for Goe1_c00030 deletion cassette

Primer extensions are presented in small letters. *B. l. = Bacillus licheniformis, B. s. = Bacillus subtilis*

References

Please consult the main manuscript for reference information.

© 2017 by the authors. Submitted for possible open access publication under the terms and conditions of the Creative Commons Attribution (CC BY) license (http://creativecommons.org/licenses/by/4.0/).
